# Mortality of acute poisoning and its predictors in Ethiopia: A systematic review and meta-analysis

**DOI:** 10.1016/j.heliyon.2024.e29741

**Published:** 2024-04-16

**Authors:** Animut Takele Telayneh, Samuel Derbie Habtegiorgis, Molla Yigzaw Birhanu, Bickes Wube Sume, Temesgen Ayenew, Getnet Gedif, Bekalu Endalew, Dejenu Tsegaye, Getasew Yirdaw, Kalkidan Worku Mitiku, Frehiwot Molla, Nurilign Abebe Moges, Getachew Mullu Kassa

**Affiliations:** a Department of Public Health, College of Medicine and Health Sciences, Debre Markos University, Debre Markos, Ethiopia; b Department of Anatomy, College of Medicine and Health Sciences, Debre Markos University, Debre Markos, Ethiopia; c Department of Nursing, College of Medicine and Health Sciences, Debre Markos University, Debre Markos, Ethiopia; d Department of Environmental Health, College of Medicine and Health Sciences, Debre Markos University, Debre Markos, Ethiopia; e Ethiopian Public Health Institute, Addis Ababa, Ethiopia

**Keywords:** Acute poisoning, Mortality, Predictors, Ethiopia

## Abstract

**Introduction:**

Poison is defined as any chemical that has the potential to affect or harm human physiology due to its chemical activity. Poisoning is becoming a major preventable public health issue in many countries, including Ethiopia. There is a variation in acute poisoning mortality among the existing evidence in Ethiopia. This study aims to determine the pooled mortality rate from acute poisoning and its predictors in Ethiopia.

**Methods:**

We searched available evidence of acute poisoning mortality in databases such as PubMed, Hinari, Cochrane, ScienceDirect, and other search engines. Using the Microsoft Excel data extraction form, three authors independently extracted all relevant data. The Higgins I^2^ test statistics were used to examine heterogeneity among included studies A random-effects model was used to analyze the pooled estimates and predictors in Stata MP version 17.

**Results:**

We retrieved 2685 relevant records from different database sources, and after screening, 21 studies (17 published and 4 unpublished) were included. The pooled mortality rate for acute poisoning was 4.69(95 % CI: 3.69, 5.69 I^2^ = 94.7 %). The most common poisoning agents are organophosphate (29.9 %), household cleansing agents (17.5 %), and pharmaceuticals/medications (9.3 %). The majority of poisoning cases were intentional poisoning committed suicide. Poisoning cases in rural areas [RR: 3.98(95 % CI: 1.41, 11.25)] and delayed arrival times [RR: 2.90(95 % CI: 1.45, 5.84)] were identified predictors of mortality.

**Conclusions:**

In this study, the pooled mortality from acute poisoning was 4.69 %. Poisoned cases from rural areas and delayed arrival times to the hospital were predictors of mortality. To prevent mortality, healthcare professionals should give special attention to rural residents and delayed arrival of poison cases. To control this avoidable death, poison control centers should be strengthened, and other preventive measures implemented at the national level.

## 1 Introduction

Poison is defined as any chemical that can alter or harm humans’ normal physiology through general or local cell damage or death by its chemical activity [[1], [2], [3]]. Poisoning is becoming a major public health problem in many countries [[4], [5], [6]]. It is the most common reason for a visit to the emergency department, with severe consequences such as morbidity, hospitalization, mortality, and financial crisis [[1], [2], [3],[7], [8], [9]]. The incidence of poisoning is not accurately known due to a paucity of records and unreported cases [2,10,11]. Globally, around 370,000 and 193,469 deaths are recorded each year due to intentional and unintentional poisonings, respectively. Low and middle-income countries account for (84 %) of poisoning incidents [12,13]. Unintentional poisoning is the most common in children under 5 years, while intention poisoning increases between 13 and 19 years [[14], [15], [16]].

The poisoning mortality rate in India ranges from 0.99 % to 5 % [[16], [17], [18]], 5.35 % in South Korea [19], and 5.1 % in Nepal [20]. According to the World Health Organization (WHO), unintentional poisoning caused an estimated 16,500 deaths in sixteen African countries. It ranges from 0.3 in Mauritius to 8.1 in Mozambique per 100,000 people, but this is underestimated the actual figure due to unreported cases [5]. Poisoning mortality rates in Ethiopia were 27.6 % in Mettu [2], 5.8 % in Jimma [21], 7.1 % in Wollega [7], 0.3 % in Gondar [22], and 62 % in Bahir Dar [23]. Pesticide poisoning kills about 300,000 people per year around the world [2,24]. Pesticide self-poisoning accounts for approximately 20 % of all suicides worldwide and is mostly linked to growing widespread use in agricultural areas in low and middle-income countries [4,9,25,26].

The pattern of poisoning varies between countries and geographical areas; pesticides, kerosene, household cleaning products, traditional medicines, and natural toxins are the most common poisoning agents [3,5,12,13]. In countries with an agrarian economy, organophosphates are the most common farming insecticides, herbicides, and pesticide agents that remain as poisoning agents [12,27]. It also accounts for the majority of suicide attempts in many under-developing countries [27,28]. According to evidence, unmet expectations, changing or breaking down of local cultures, chronic disease states, business loss, love failure, illiteracy, younger age, gender, marital status, and living alone are the reasons for intentional poisoning [2,12,17,29]. However, weak regulatory activities, residence, sex, arrival time to the hospital, length of hospital stay, management type, mode of poisoning, and age were all identified as risk factors for poisoning mortality [1,3,7,[30], [31], [32]]. Poison control centers are important measures to reduce morbidity and mortality from poisoning because they facilitate poisoning diagnosis, treatment, and preventive measures [5,9,10,33]. Ethiopia lacks a well-organized poison control center, making it extremely difficult to obtain primary data on poisoning cases and mortalities before reaching the hospitals [8,33]. There is substantial variation in evidence of acute poisoning mortality between regions in Ethiopia [2,21,34]. Additionally, no systematic review and meta-analysis has been done to enhance the quality and consistency of the evidence. The purpose of this systematic review and meta-analysis was to determine the pooled mortality of acute poisoning and its predictors in Ethiopia using available evidence. This study will help health professionals, policymakers, programmers, and planners establish and enforce case management, and control strategies, as well as implement effective interventions, to reduce the preventable mortality burden in Ethiopia.

## 2 Materials and methods

### 2.1 Search strategy

Three authors (ATT, SDH, and BE) independently searched PubMed, Hinari, Cochrane, ScienceDirect, Google Scholar, and other search engines for articles reporting poisoning-related mortalities (**S1 Table**). Our search was extended by doing hand searches for gray literature and retrieving reference lists of eligible articles. The literature search was completed in March 2023. The Condition, Context, and Population (CoCoPop) criteria were employed to search the databases using a comprehensive searching strategy that included common Boolean words such as Mortality OR Outcome OR Acute poisoning OR poisoning OR epidemiology OR associated factors OR predictors AND Ethiopia OR Federal Democratic of Ethiopia AND all poisoned cases. We presented this systematic review and meta-analysis by the Preferred Reporting Items for Systematic Reviews and Meta-Analysis (PRISMA) guidelines [35]. Publications with incomplete data will be rectified by contacting the corresponding author. It was then exported to EndNote version 20.4 software to manage duplicated articles.

### 2.2 Protocol registration

This systematic review and meta-analysis were registered under the PROSPERO 2023 ID: CRD42023404236. The protocol was published by the National Institute for Health Research University of York PROSPERO International Prospective Register of Systematic Reviews. Some amendments were made to the information provided at registration or in the protocol.

### 2.3 Eligibility criteria

**Inclusion criteria:** Before the search began, eligibility criteria were determined. We included all published and unpublished observational studies that reported acute poisoning mortality/death. This review included studies conducted in Ethiopia and published in English.

**Exclusion criteria:** Case reports/series, systematic reviews, books, and guidelines were all excluded. Articles reporting no mortality from acute poisoning were not included. Moreover, articles that were not fully accessible, despite at least two email contacts with the primary authors were removed due to the difficulties of assessing the quality of articles without full text.

### 2.4 Data extraction

Three different authors (ATT, SDH, and BE) searched articles, (ATT, MYB, and KW) independently screened the retrieved articles using their titles and abstract, and (ATT, TA, and GG) extracted data using a predetermined Microsoft Excel spreadsheet format. The data extraction format includes the first author, publication year (which was used as the study year for unpublished articles), study area, participant age, sample size, response rate, intentional poisoning rate, and mortality rate. Any disagreements among authors during the extraction were resolved by discussion and mutual agreement with the help of a fourth author (DT).

### 2.5 Outcome measurement

In this study, estimating mortality rates and identifying predictors are the outcomes reported from this systematic review and meta-analysis. The primary outcome is the pooled acute poisoning mortality rate. This is computed by the number of mortalities divided by the number of acute poisoning cases included in the studies (sample size) and multiplied by 100. The relative risk (RR) of predictor variables was calculated using the two-by-two tables with binary outcomes of residence (rural/urban), arrival time to the hospital (≤1 h/>1 h), length of hospital stay (≤48 h/>48 h), and sex (male/female).

### 2.6 Quality assessment

The Newcastle Ottawa Scale for cohort studies was used to assess the quality of the included studies. The tool has three main dimensions: the first dimension addresses the assessment of the selection of the exposed and non-exposed groups using the representativeness of the exposed group, the selection of the non-exposed cohort, ascertainment of exposure, and demonstration of the outcome of interest, which was not present at the beginning of the study. The second dimension assesses the comparability of the cohorts based on the design or analysis. The third dimension examines the quality of outcome addressing through assessment of the outcome, ways to follow-up long enough for the outcome to occur, and adequacy of follow-up of cohorts [36,37]. The study gets a score of ≥5 stars from a total of 9 scores considered as high quality (S2 and 3 Tables).

### 2.7 Statistical data analysis

The data were extracted using Microsoft Excel and then exported to Stata MP version 17 software meta-analysis package for analysis. We calculate the effect size for individual studies by generating incidence to each study using mortalities divided by sample size and multiplied by 100 (incidence = mortalities/sample size *100). Then the incidence is transformed to standard error using incidence multiplied by after (incidence subtracted from 100) and divided by sample size (standard error incidence = incidence*(100-incidence)/sample size). We used tables and forest plots to present the results. The pooled estimate of the meta-analysis, together with a 95 % confidence interval (CI), was presented using a forest plot. Heterogeneity between studies was assessed by the calculated p-value of Higgins I^2^-test statistics; values 25 %, 50 %, and 75 % indicated low, moderate, and high heterogeneity testing, respectively [38]. To assess publication bias, a funnel plot visual inspection and Egger's objectivity test were used with slope intercept p-value less than 0.05 [39,40]. A random-effect model was used as a method of analysis to test results in the presence of heterogeneity [41,42]. In addition, to execute the heterogeneity among the included studies, subgroup analysis was done based on the group's regions of country, age, follow-up period, sample size, publication year, proportion of participants' response, and intentional poisoning.

## 3 Results

### 3.1 Description of selection procedure eligible articles

**Fig. 1:**
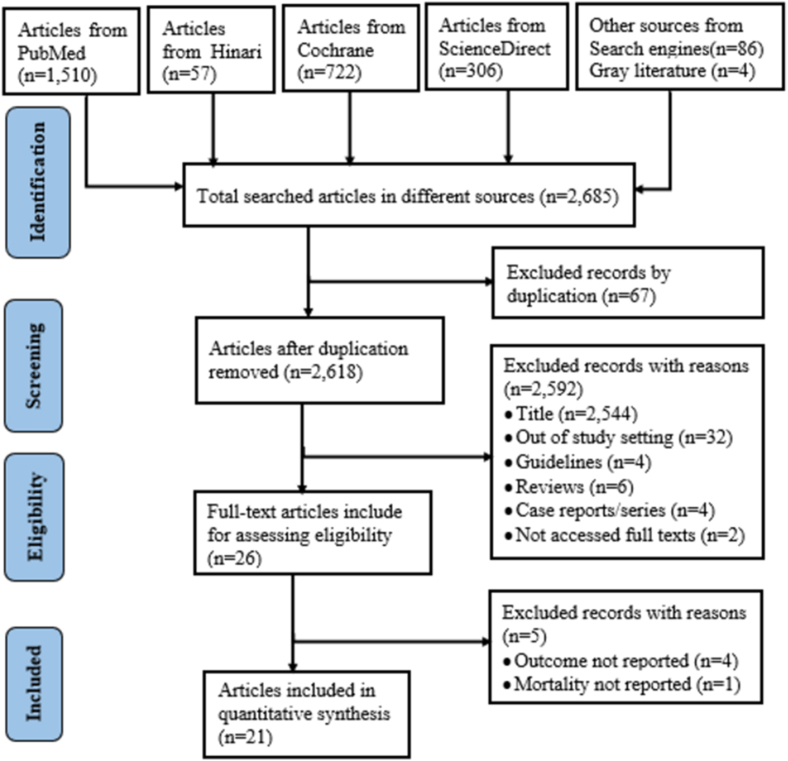
Flow diagram to show the article selection procedure for systematic review and meta-analysis.

We retrieved 2685 relevant records from databases PubMed, Hinari, Cochrane, ScienceDirect, and other search engine sources. After excluding duplications, 2618 articles were screened. Finally, 21 articles (17 published and 4 unpublished) were included in this systematic review and meta-analysis (Fig. 1).

### 3.2 Characteristics of the included studies

In this study, 21 studies met the inclusion criteria and included in systematic review and meta-analysis among those only four studies [31,32,43,44] were unpublished. All of the studies were conducted in four regions: nine in Amhara [3,22,23,30,31,43,[45], [46], [47]], five in Addis Ababa [13,32,44,48,49], six in Oromia [1,2,7,21,50,51], and the last one study in Harar [52], with a total of 4196 respondents. The included studies’ follow-up period ranges from 1.8 months in Dessie [3] to 6 years in Jimma [51]. The smallest and largest estimated sample sizes were 76 in Mettu [2] and 998 in Addis Ababa [49], respectively. The lowest response rate in the included study was 59.3 % [49]. The mortality rate in the included studies ranges from 0.3 % in Gondar [22] to 62 % in Bahir Dar [23]. The proportion of intentional poisoning ranges from 31 % in Gondar [22] to 100 % in Bahir Dar [43,46] (Table 1). The majority of the included studies do not provide distinct arrival timings and lengths of hospital stays. Despite this, four studies reported hospital arrival times of less than 30 min [21,32,43,46], with only one study reporting an arrival time of 96 h following poisoning exposure [32]. Similarly, only 9 studies reported the length of hospital stay; of these, the minimum length of stay was one day [13], and the maximum was 11 days [46].

**Table 1:** Characteristics of 21 studies reported the mortality of acute poisoning in Ethiopia.

Authors	Pub/year	Region (area)	Ages	Follow-up period in months	Samples	Response rate (%)	Intentional poisoning (%)	Mortality rate (%)
Adinew et al. [22]	2017	Amhara (NGZ)	All ages	52	543	63.4	31	0.3
Bereda et al. [2]	2021	Oromia (Mettu)	All ages	13	76	100	64.5	27.6
Bogale et al. [46]	2021	Amhara (BDR)	All ages	30	141	89	100	31.2
Chala et al. [50]	2015	Oromia (Adama)	All ages	24	292	100	36.6	1.37
Desalew et al. [48]	2011	Addis Ababa	Adults	24	116	100	96.6	8.6
Eyasu et al. [49]	2017	Addis Ababa	Adults	60	998	59.3	85.5	1.2
Getie et al. [3]	2020	Amhara (Dessie)	All ages	1.8	147	81.6	64.2	6.6
Molla et al. [30]	2022	Amhara (Gondar)	Children	48	103	79.6	24.4	8.5
Nigussie et al. [52]	2022	Harar	All ages	60	175	85.2	51.3	16.7
Shumet et al. [23]	2022	Amhara (BDR)	All ages	12	121	100	98.3	62
Tefera et al. [1]	2020	Oromia (Ambo)	All ages	14.5	134	100	76.9	1.5
Teklemariam et al. [21]	2016	Oromia (Jimma)	All ages	24	110	93.6	50.5	5.8
Woyessa et al. [7]	2020	Oromia (Wollega)	All ages	10	211	100	46.45	7.1
Zemedie et al. [13]	2021	Addis Ababa	All ages	12	98	100	98	10.2
Adinew et al. [45]	2016	Amhara (Gondar)	All ages	52	233	100	57.5	0.43
Dessie et al. [32]	2021	Addis Ababa	Adults	36	218	83.9	92.3	13.7
Melese et al. [44]	2018	Addis Ababa	All ages	12	306	93.2	88.5	8.4
Getnet H [43]	2022	Amhara (BDR)	Adults	12	268	100	100	20.9
Endayehu et al. [47]	2019	Amhara (DT)	All ages	36	102	100	91.2	18.6
Ahmed et al. [51]	2018	Oromia (Jimma)	All ages	72	236	100	76.7	6.4
Mengistie Y [31]	2022	Amhara (BDR)	Adults	24	304	100	88.2	30.6

*Note: NGZ: North Gondar Zone; BDR: Bahir Dar; DT: Debre Tabor.

### 3.3 The magnitude of poisoned cases by type of poisoning agent

In the 21 studies that were included, 15 types of known poisoning agents were reported. Three most common poisoning agents reported were organophosphate 1255(29.9 %), bleach/household cleaning agents 735(17.5 %), and pharmaceuticals/medications 390(9.3 %). Hydrogen peroxide, snake venom, and herbal/traditional medicines account for the least poisoning cases (Table 2).

**Table 2:** Number of poisoned cases by type of poisoning agents in Ethiopia.

S/no	Types of poisoning agents	Number of poisoned cases (N)	Percent (%)
1	Organophosphates	1255	29.9
2	Bleach/household cleaning agent	735	17.5
3	Pharmaceuticals/medications	390	9.3
4	Other/unknown chemicals	285	6.8
5	Rodenticides	262	6.2
6	Aluminum phosphate	224	5.3
7	Alcohol	200	4.8
8	Organ chemicals/pesticides	197	4.7
9	Rate poison	187	4.5
10	Carbon monoxide	176	4.2
11	Food poisoning	114	2.7
12	Kerosene/Benzine	65	1.5
13	Herbal/Traditional medication	58	1.4
14	Snake Venom	39	0.9
15	Hydrogen peroxide	9	0.2

### 3.4 Meta-analysis

**Fig. 2:**
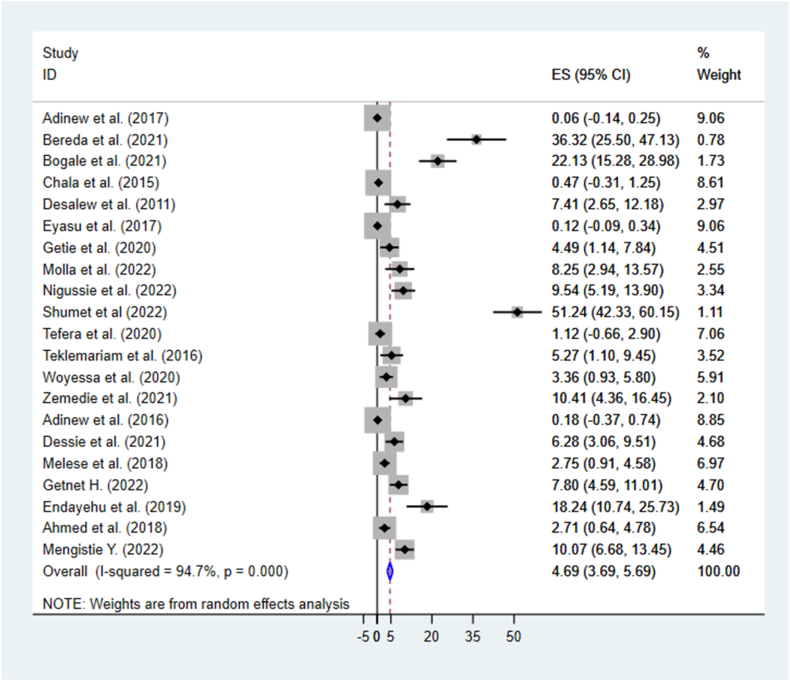
The pooled mortality rate of acute poisoning in Ethiopia.

The pooled mortality rate from acute poisoning in this systematic review and meta-analysis was 4.69(95 % CI: 3.69, 5.69, I^2^ = 94.7 %). The I^2^ test revealed high heterogeneity among included studies; the model used random effect (I^2^ = 94.7 %, P =<0.001) (Fig. 2).

### 3.5 Subgroup analysis

**Fig. 3:**
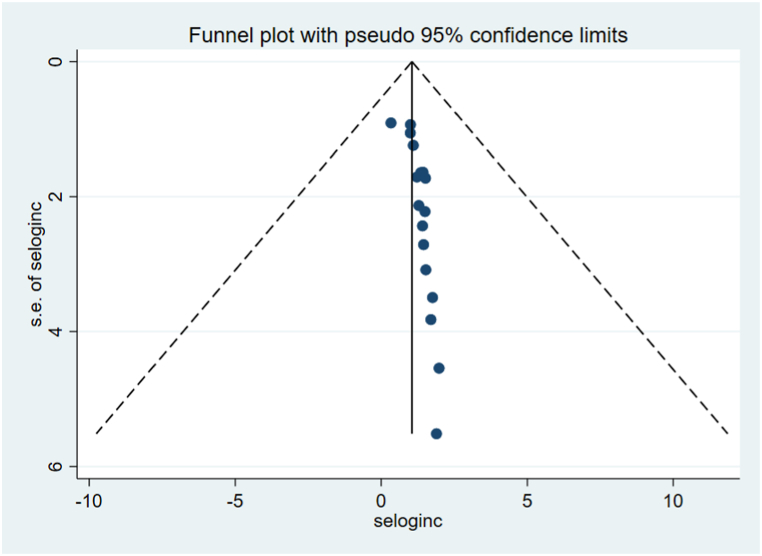
Funnel plot analysis of included studies for mortality of acute poisoning in Ethiopia.

**Fig. 4:**
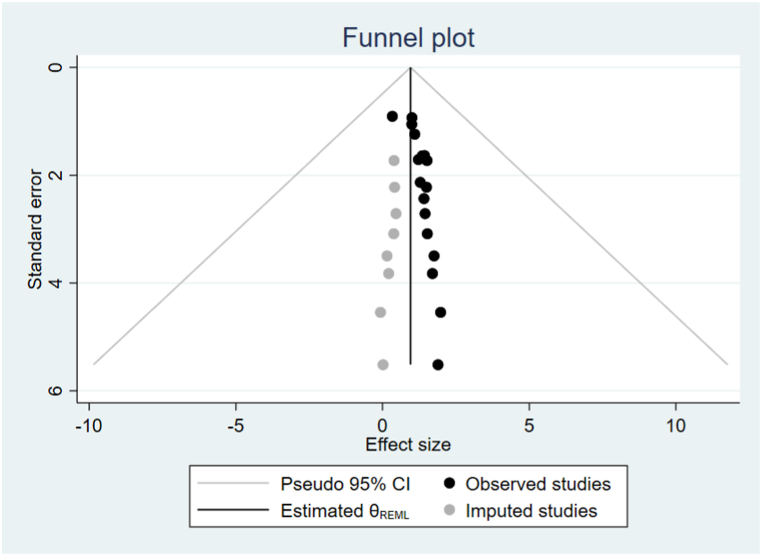
Fill and trim funnel plot analysis for mortality of acute poisoning in Ethiopia.

In this systematic review and meta-analysis, we investigated study heterogeneity used subgroup analysis. The highest mortality rate was observed in Amhara 9.30(95 % CI: 6.78, 11.82), followed by Addis Ababa 4.61(95 % CI: 1.37, 7.84). Similarly, the mortality rate was higher in the last 5 years of publication 11.24(95 % CI: 7.79, 14.69), and above 72.3 % of intentional poisoning cases 9.87(95 % CI: 6.52, 13.21) (Table 3). To examine publication bias between included studies, funnel plot visual inspection, and it appears asymmetric distribution of studies shows the presence of publication bias (Fig. 3) This was also checked by performing the Eggers objectivity test, which indicates the presence of publication bias (p < 0.001). Hence, trim and fill analysis was used to alter the pooled mortality rates of acute poisoning (Fig. 4).

**Table 3:** Subgroup analysis of mortality rate of acute poisoning in Ethiopia.

Variable	Characteristics	No. studies	Study participants	Mortality rate at (95 % CI)	I^2^ tests (%)
Region	Amhara	9	1699	9.30(6.78, 11.82)	96.9
	Oromia	6	1052	4.23(1.34, 7.12)	90.5
	Addis Ababa	5	1295	4.61(1.37, 7.84)	90.3
	Harar	1	150	9.54 (5.19, 13.90)	–
Age groups	All ages	15	2651	5.93(4.31, 7.56)	95.2
	Adults only	5	1463	6.20(1.21, 11.18)	94.8
	Children only	1	82	8.25(2.94, 13.57)	–
Follow-up period	≤30 months	13	2274	10.36(6.91, 13.82)	95.3
	>30 months	8	1922	1.29(0.51, 2.08)	90.0
Sample size	≤234	14	1854	11.44(7.72, 15.17)	95.5
	>234	7	2342	1.50(0.72, 2.29)	91.4
Publication year	2018–2022^a^	15	2516	11.24(7.79, 14.69)	93.7
	2017 and before	6	1680	0.24(0.12, 0.60)	68.9
Response rate	<100 %	9	2005	2.70(1.67, 3.73)	92.1
	100 %	12	2191	8.85 (6.13, 11.57)	95.8
Intentional poisoning	≤72.3 %	9	2090	2.85(1.51, 4.20)	91.1
	>72.3 %	12	2994	9.87(6.52, 13.21)	96.2

Note.

a Used publication year as study year for unpublished articles.

### 3.6 Predictors of mortality acute poisoning

**Fig. 5:**
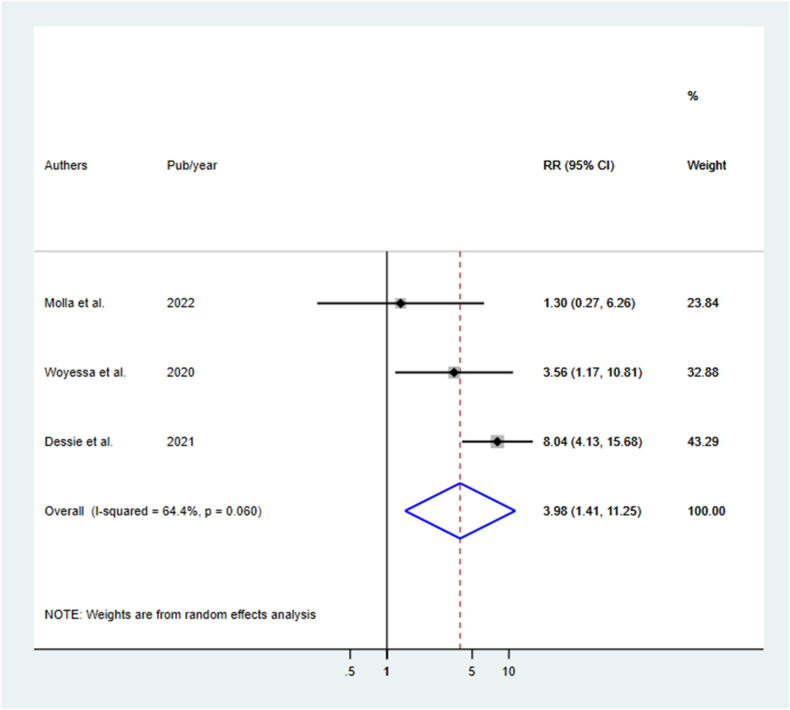
Predictors of mortality of acute poisoning in Ethiopia by the residence of the participants.

**Fig. 6:**
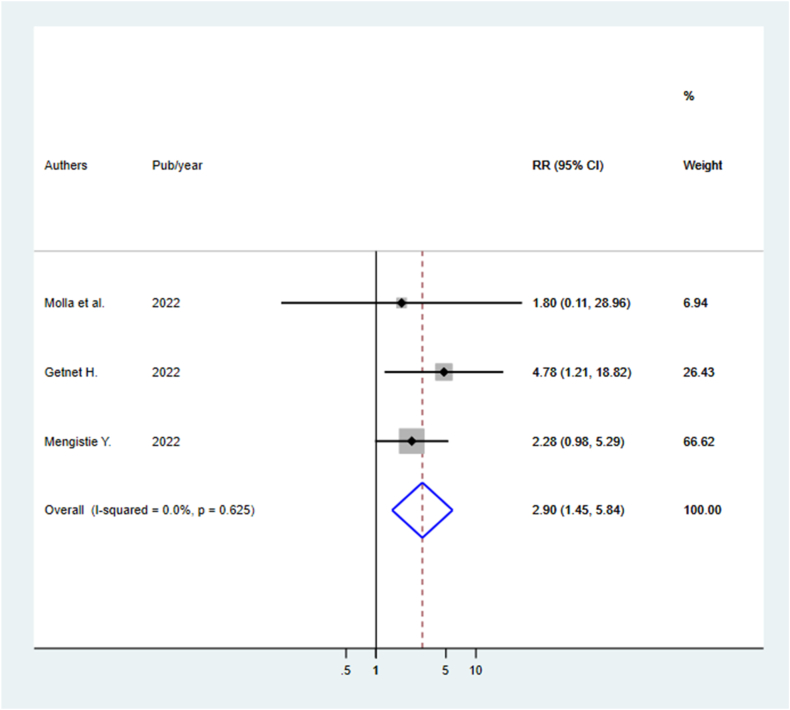
Predictors of mortality of acute poisoning in Ethiopia by arrival time after poisoning exposure.

In this systematic review and meta-analysis, participants’ residence and arrival time at the hospital were identified as predictors of acute poisoning mortality. Three studies with a sample size of 532 reported the association between residence and mortality from acute poisoning [7,30,32]. Poisoned cases from rural residents were 4 times more at risk of mortality compared to counterparts of urban residents [RR: 3.98(95 % CI: 1.41, 11.25)] (Fig. 5). Similarly, delayed arrival time to the hospital after poison exposure was a statistical predictor of mortality reported in three studies with the sum of sample sizes 675 [30,31,43]. Poisoned cases who arrived at the hospital after 1 h of poisoning exposure to the hospital were nearly three times more at risk of mortality compared who arrived within 1 h of exposure [RR: 2.90(95 % CI: 1.45, 5.84)] (Fig. 6). However, length of hospital stay from three studies [1,30,43] and sex from two studies [1,31] were not statistically significant predictors of mortality.

## 4 Discussion

Acute poisoning is becoming a public health problem and the major cause of emergency department hospitalization, morbidity, and mortality globally [[4], [5], [6],^11^,^12^,^53^,^54^]. The majority of them are deliberate self-poisoning committed to suicide, which is the most preventable mortality [4,5,28,33,54,55]. This systematic review and meta-analysis tried to assess the pooled acute poisoning mortality rate in Ethiopia. In this study, the pooled mortality rate of acute poisoning was 4.69 (95 % CI: 3.69, 5.69, I^2^ = 94.7 %). This finding is consistent with previous evidence reported 4 % in Southern and 5 % in South-Eastern India [16,18], and 5.1 % in Nepal [20]. This study was higher than the systematic review and meta-analysis study conducted 0.8 % in Iran [56]. The previous study's inclusion of a different study population in pediatrics may have the possible reason for to decrease in mortality rates. This finding is higher in the single studies reported: 1.1 % in Saudi Arabia [57], 0.99 % in Karnataka and 2.4 % in Bengaluru India [17,58], and 2.6 % in Taiwan [59]. The difference might be attributed to the study setting, design, and sample size. Previous studies were carried out with a high level of knowledge, awareness, and use of technology, large sample sizes, and the presence of well-functioning poison control strategies.

This finding is lower than those reported in systematic reviews and meta-analyses: 7 % in Thailand [55], 27 % [60] in Iran, and 13.7 % in pesticide poisoning globally [61]. This variation might be due to differences in the study setting, which resulted in strengthened poison information and control centers for early tracking mortalities, the availability of new poisoning agents, and increased accessibility to pharmaceuticals in the previous studies. The study population's awareness level and usage of technology have also played a pivotal role in reporting poisoning cases and associated health outcomes in Thailand and Iran [55,62]. In Ethiopia, there is weak regulatory activity, and the increased utilization of agricultural chemicals such as herbicides, pesticides, and rodenticides has boosted poisoning incidents recently [25,26,33,[63], [64], [65]]. However, the current study lacks the ability to track unreported poisoning cases and mortality rates, which may underestimate the national incidence of acute poisoning mortality. Furthermore, this is also lower single studies reported ranges from 6.4 % to 16.5 % [27,[66], [67], [68], [69], [70]] in different regions, including poison information centers in India, 8.3 % in Bangladesh [71], and 20.8 % in Korea [19]. This might be due to differences in study settings and incomparability of single study mortality with the pooled effect size resulting from the mortality of multiple studies.

Organophosphates 1255(29.9 %), bleach/household cleaning agents 735(7.5 %), pharmaceuticals/medications 390(9.3 %), other/unknown chemicals 285(6.8 %), and rodenticides 262(6.2 %) were the most common five poisoning agents reported in this study. This is supported by previous systematic reviews in Ethiopia, Iran, and the multi-country developing world [56,[72], [73], [74]]. There are also no significant variations between the single studies' reported in Iran, which found that pharmaceutical drugs, pesticides, and insecticides are the leading causes of poisoning [62,75]. Pesticides followed by household agents, and pharmaceutical products are the major causes of poisoning in Thailand [55]. Similarly, other studies’ findings are consistent with this finding reported in India, Bangladesh, and multicounty studies [18,58,[69], [70], [71],[76], [77], [78]].

The majority of the studies included in this systematic review and meta-analysis study reported that the reason for poisoning was intentional poisoning for self-suicidal attempts, ranging from 24.4 % [30] to 100 % [43,46]. This is consistent with the previous evidence conducted in Ethiopia, South Africa, Thailand, India, Korea, and Nepal [19,20,55,67,[72], [73], [74],^76^,^78^,^79^].

The predictors of acute poisoning mortality are not well addressed in published articles (most of the existing evidence is descriptive studies). In this systematic review and meta-analysis study, rural residents’ acute poisoning cases were identified as predictors of mortality compared to urban residents. This finding is consistent with the study conducted in Sira Lanka and Zambia [80,81]. Rural dwellers are illiterate, have low health-seeking behaviors, are inaccessible to health facilities due to transportation problems, and have poor availability of health resources in the nearby health facilities. This might be the possible reason for the delay in seeking poisoned treatment, increasing the likelihood of severe complications and major organ failures that lead to mortality. Hospital arrival time following poisoning exposure was a statistical predictor of acute poisoning mortality. This is consistent with previous evidence reported in different studies in India and Nepal [18,20,67]. The clinical management of poisoning and the outcome of treatment are determined by the timing of arrival after exposure. Delayed arrival time to the hospital may increase mild to severe health conditions including headache, vomiting, tachypnea, tachycardia, altered consciousness, comma, abdominal pain, bleeding, epigastric pain, and major organ failure, which can lead to mortality [24,54,67,82,83]. Nevertheless, length of hospital stay and sex were not statistical predictors for acute poisoning mortality in this systematic review and meta-analysis. This evidence is not in line with previous findings in Ethiopia and Sri Lanka [73,84]. This might be due to the small sample size of individual studies, as well as the small number of articles that reported predictors of mortality, which could obscure the association when the effect size of multiple studies is pooled.

In subgroup analysis revealed that Amhara region, adults, follow-up period 2.5 years and less, small sample size, high response rate, and intentionally poisoned groups had higher mortalities. This is attributable to the region's huge number of studies, the higher likelihood of suicide attempts in adults, the fact that deliberate self-poisoning cases are self-suicidal, and the traceability of current patient charts. All of these reasons may increase acute poisoning mortality rates compared to counterparts in this study. In general, hospital arrival time, residence, and length of hospital stay are clinically relevant variables for preventing acute poisoning mortality and require great attention in clinical settings and the community as a whole.

## 5 Limitations of the study

Due to a lack of available evidence, this study was unable to cover all parts of the country.

## 6 Conclusions

In this systematic review and meta-analysis, preventable acute poisoning mortality was 4.69 %. Rural dwellers and delayed arrival times to the hospital were identified as predictors of acute poisoning mortality. Health promotion and education activities are encouraged to prevent this looming public health problem. Healthcare professionals devote special attention to acute poisoning cases from rural areas and those with delayed hospital arrival times. All higher-level official bodies should have well-functioning and strengthened poison information and control centers. Implement immediate interventions and other national-level initiatives to prevent such preventable mortalities. Moreover, further multicenter prospective studies with large sample sizes should be conducted to address unreported cases, chart incompleteness, regions with no prior evidence, and potential predictors of acute poisoning mortality.

## Ethics approval and consent to participate

Not applicable.

## Consent to publication

Not applicable.

## Availability of data and materials

Not applicable or all data used in this study was presented in this document.

## Funding statement

This research has not received specific funding.

## CRediT authorship contribution statement

**Animut Takele Telayneh:** Writing – review & editing, Writing – original draft, Visualization, Software, Resources, Project administration, Methodology, Investigation, Funding acquisition, Formal analysis, Data curation, Conceptualization. **Samuel Derbie Habtegiorgis:** Writing – review & editing, Writing – original draft, Validation, Software, Methodology, Investigation, Formal analysis, Data curation. **Molla Yigzaw Birhanu:** Writing – review & editing, Writing – original draft, Validation, Supervision, Software, Investigation, Data curation. **Bickes Wube Sume:** Writing – review & editing, Writing – original draft, Supervision, Software, Methodology, Investigation, Formal analysis. **Temesgen Ayenew:** Writing – review & editing, Writing – original draft, Visualization, Validation, Software, Resources, Methodology, Investigation. **Getnet Gedif:** Writing – review & editing, Writing – original draft, Validation, Supervision, Methodology, Investigation, Formal analysis, Data curation. **Bekalu Endalew:** Writing – review & editing, Writing – original draft, Visualization, Validation, Methodology, Investigation, Formal analysis, Data curation. **Dejenu Tsegaye:** Writing – review & editing, Writing – original draft, Visualization, Validation, Supervision, Software, Methodology, Formal analysis, Data curation. **Getasew Yirdaw:** Writing – review & editing, Writing – original draft, Software, Methodology, Investigation, Formal analysis, Data curation. **Kalkidan Worku Mitiku:** Writing – review & editing, Writing – original draft, Supervision, Software, Methodology, Formal analysis, Data curation. **Frehiwot Molla:** Writing – review & editing, Visualization, Validation, Software, Methodology, Formal analysis, Data curation. **Nurilign Abebe Moges:** Writing – review & editing, Writing – original draft, Software, Methodology, Formal analysis, Data curation. **Getachew Mullu Kassa:** Writing – review & editing, Writing – original draft, Software, Methodology, Formal analysis, Data curation.

## Declaration of competing interest

The authors declare the following financial interests/personal relationships which may be considered as potential competing interests:Animut Takele reports administrative support was provided by Debre Markos University. Animut Takele reports a relationship with Debre Markos University that includes: employment. Animut Takele has patent pending to no. No If there are other authors, they declare that they have no known competing financial interests or personal relationships that could have appeared to influence the work reported in this paper.
