# Supplementary material for: Mortality of acute poisoning and its predictors in Ethiopia: A systematic review and meta-analysis
Source: Heliyon. 2024 Apr 16;10(8):e29741. doi: 10.1016/j.heliyon.2024.e29741 (PMC11046229; doi:10.1016/j.heliyon.2024.e29741)
Supplement: Multimedia component 1 [file mmc1.docx]

| Search number | Quarries | Results |
| --- | --- | --- |
| 1 | ((((((((((((poisoning) OR (acute poisoning)) OR (reasons)) OR (mode)) OR (organophosphate)) OR (phenobarbitone)) OR (bleach)) OR (household agents)) OR (intentional self-harm poisoning))) OR (poisoning[MeSH Terms])) OR (organophosphates[MeSH Terms])) OR (phenobarbital[MeSH Terms]) | 1,272,615 |
| 2 | ((Ethiopia) OR (Federal Democratic republic of Ethiopia)) OR (Ethiopia[MeSH Terms]) | 35,175 |
| 3 | ((((((((((((((Pediatric) OR (Children)) OR (Youth)) OR (Adult)) OR (Men)) OR (Women)) OR (Male)) OR (Female)) OR (Pregnancy)) OR (Children[MeSH Terms])) OR (Adolescent[MeSH Terms])) OR (Adult[MeSH Terms])) OR (Men[MeSH Terms])) OR (Women[MeSH Terms])) OR (Pregnancy[MeSH Terms]) | 15,534,818 |
| 4 | ((((((((((((((poisoning) OR (acute poisoning)) OR (reasons)) OR (mode)) OR (organophosphate)) OR (phenobarbitone)) OR (bleach)) OR (household agents)) OR (intentional self-harm poisoning)) ) OR (poisoning[MeSH Terms])) OR (organophosphates[MeSH Terms])) OR (phenobarbital[MeSH Terms])) AND (((Ethiopia) OR (Federal Democratic republic of Ethiopia)) OR (Ethiopia[MeSH Terms]))) AND (((((((((((((((Pediatric) OR (Children)) OR (Youth)) OR (Adult)) OR (Men)) OR (Women)) OR (Male)) OR (Female)) OR (Pregnancy)) OR (Children[MeSH Terms])) OR (Adolescent[MeSH Terms])) OR (Adult[MeSH Terms])) OR (Men[MeSH Terms])) OR (Women[MeSH Terms])) OR (Pregnancy[MeSH Terms])) | 1,510 |
