# Supplementary material for: Mortality of acute poisoning and its predictors in Ethiopia: A systematic review and meta-analysis
Source: Heliyon. 2024 Apr 16;10(8):e29741. doi: 10.1016/j.heliyon.2024.e29741 (PMC11046229; doi:10.1016/j.heliyon.2024.e29741)
Supplement: Multimedia component 2 [file mmc2.docx]

| Author | Pub/year | Selection  (4 stars) | Comparability  (2 stars) | Outcome  (3stars) | Total score (9 stars) |
| --- | --- | --- | --- | --- | --- |
| Adinew et al. | 2017 | *** | - | ** | 5 |
| Bereda et al. | 2021 | ** | - | ** | 4 |
| Bogale et al. | 2021 | ** | - | ** | 4 |
| Chala et al. | 2015 | *** | - | ** | 5 |
| Desalew et al. | 2011 | ** | - | * | 3 |
| Eyasu et al. | 2017 | *** | * | ** | 6 |
| Getie et al. | 2020 | ** | - | * | 3 |
| Molla et al. | 2022 | ** | * | * | 4 |
| Nigussie et al. | 2022 | ** | * | ** | 5 |
| Shumet et al | 2022 | ** | - | * | 3 |
| Tefera et al. | 2020 | ** | * | * | 4 |
| Teklemariam et al. | 2016 | ** | - | * | 3 |
| Woyessa et al. | 2020 | ** | * | * | 4 |
| Zemedie et al. | 2021 | ** | - | * | 3 |
| Adinew et al. | 2016 | ** | - | ** | 4 |
| Dessie et al. | 2021 | ** | * | ** | 5 |
| Melese et al. | 2018 | ** | * | ** | 5 |
| Getnet H. | 2022 | ** | * | ** | 5 |
| Endayehu et al. | 2019 | ** | - | ** | 4 |
| Ahmed et al. | 2018 | *** | - | *** | 6 |
| Mengistie Y. | 2022 | *** | * | ** | 6 |
